# Supplementary material for: Advance directives prior to COVID-19 diagnosis in a United States national healthcare system
Source: PLoS One. 2025 Dec 19;20(12):e0338825. doi: 10.1371/journal.pone.0338825 (PMC12716786; doi:10.1371/journal.pone.0338825)
Supplement: S1 Table — (DOCX) [file pone.0338825.s001.docx]

|  | All +COVID-19 (%; n=422,028) | Proportion with Advance Directive (%; n=67,970) |
| --- | --- | --- |
| Geographic Region |  |  |
| New England | 3.2 (13,386) | 22.8 (3,051) |
| New York | 5.9 (25,032) | 18.5 (4,635) |
| Mid-Atlantic | 9.3 (39,355) | 13.9 (5,462) |
| Southeast | 26.0 (109,928) | 14.0 (15,375) |
| Midwest | 13.3 (56,205) | 18.7 (10,494) |
| South Central | 13.1 (55,333) | 14.7 (8,139) |
| Central | 7.3 (30,855) | 16.4 (5,073) |
| Mountains | 4.1 (17,181) | 17.2 (2,961) |
| Southwest | 15.0 (63,420) | 16.9 (10,713) |
| Northwest | 2.7 (11,333) | 18.2 (2,067) |
