# Supplementary material for: Advance directives prior to COVID-19 diagnosis in a United States national healthcare system
Source: PLoS One. 2025 Dec 19;20(12):e0338825. doi: 10.1371/journal.pone.0338825 (PMC12716786; doi:10.1371/journal.pone.0338825)
Supplement: Attachment — Submitted filename: REVIEWER COMMENTS WITH RESPONSES v10-13-2025.docx [file pone.0338825.s004.docx]

Reviewer #1: Thank you for allowing me to review this article, which sought to determine what proportion of VA patients who test positive for COVID-19 have evidence of an advance directive prior to infection.

The introduction seems adequate but very brief; I find it lacks a description of AD completion rates and major disparities in advanced care planning in the US.

*RESPONSE: We agree this is critical information, which we have moved up from the discussion to intro in the revised manuscript.*

The study design and population are adequately described, as are the measures used. The statistical analyses are presented appropriately, however in the logistic regression models, information on goodness of fit is missing (global Chi², Nagelkerke R², Hosmer-Lemeshow test, etc.). How overfitting was avoided.

*RESPONSE: We have added to the methods and results as follows.*

*Methods – To account for confounding, we examined factors associated with AD completion using multivariable logistic regression modeling guided by subject area knowledge, Akaike information criterion (AIC) and area under the curve (AUC and Nagelkerke R²).*

*Results – Multivariable models 2-5 showed better fit and discrimination with further adjustment as evidenced by decreasing AIC and increasing AUC and R² (Table 2). The biggest improvement came from addition of the VACO Index.*

|  | *1* | *2* | *3* | *4* | *5* |
| --- | --- | --- | --- | --- | --- |
| *AIC* | *356,312* | *355,218* | *354,675* | *348,218* | *344,538* |
| *AUC* | *0.649* | *0.655* | *0.658* | *0.684* | *0.697* |
| *R-Square* | *0.038* | *0.040* | *0.042* | *0.056* | *0.064* |

In Table 1 of patient characteristics, review the figures and % in some columns (Quarter, Primary Care, VACO Index, Mortality), it would also be good to know the Age (years), Median (IQR) for those who had a completed advance directive. The conclusions supported by the data.

*RESPONSE: We have added the Age (years), Median (IQR) for those who had a completed advance directive. We have clarified the column descriptions in Table 1.*

Reviewer #2: This study addresses an important and timely topic on advance directive completion among veterans with COVID 19 in the United States. The manuscript is well structured but some revisions would improve clarity, transparency and global readability. The study design should be described consistently as a retrospective cohort study in the abstract, methods and conclusions.

*RESPONSE: We have edited the manuscript to describe the study consistently as a retrospective cohort study.*

The title and abstract should specify the United States so that international readers unfamiliar with the VA system can clearly understand the setting.

*RESPONSE: We have edited the title and abstract to specify the United States.*

The abstract wording could be simplified by using phrases such as advance directive completed prior to COVID 19 diagnosis and confidence intervals should be reported in parentheses rather than brackets.

*RESPONSE: We have edited the abstract wording as suggested by the reviewer and reported confidence intervals in parentheses.*

The statistical methods require more detail including which tests were used for descriptive comparisons such as chi square or t test and a clear statement that multivariable logistic regression was used with justification for this choice.

RESPONSE: We added clarification regarding statistical analysis, noting:

*“We compared AD status by patient demographics, marital status, geographic region, healthcare utilization and severity of illness using the VACO Index, using chi-square test for categorical variables and t-test for continuous variables”*

Descriptive statistics should always specify whether values are means or medians.

*RESPONSE: Age is reported consistently as a median, as noted in the text and table. We reviewed additional results regarding descriptive statistics.*

Results need fuller explanation of the calendar quarter data already shown in Table 1 and the text should clarify whether percentages in tables and figures are row based column based or overall.

*RESPONSE: We have edited Table 1 to address the concern regarding row versus column-based percentages.*

*We added to the methods section:*

*“In exploratory analysis, we evaluated whether associations differed by calendar time. We created 3 categories: Pre-vaccine Mar-Dec 2020, Vaccine Jan-Dec 2021, and Omicron Jan-Dec 2022.”*

*We added to the results section:*

*“The proportion with AD decreased from 18.5% in Mar-June 2020 to 13.6% in Jul-September 2021, then began rising reaching 21.5% in Oct-Dec 2022 (Table 1). This is reflected in the unadjusted odds ratios 1.20 (95% Confidence Interval 1.14-1.27) in Oct-Dec 2022 in (Table 2). This is likely due to older age. In the earliest period 34% were age 70 years or higher, rising to 47% in the latest period.” and “After adjustment for age, race and marital status (Model 3) the increase in AD completion during Oct-Dec 2022 in unadjusted models was attenuated to OR 1.04 (0.98-1.09) and remained so in Model 5 OR 0.96 (0.91-1.02). We found similar associations across the Pre-vaccine, Vaccine, and Omicron eras (data not shown).”*

The flow of the results could be improved by first presenting descriptive findings then unadjusted associations and finally multivariable models.

*RESPONSE: We reviewed the flow of results to ensure descriptive/univariate findings are presented prior to multivariable models.*

The discussion and limitations section should be expanded to note residual confounding and unmeasured variables which are common in observational studies.

*RESPONSE: We have edited the text to note these limitations.*

Policy implications should be made stronger by discussing how the Veterans Health Administration and the broader United States health system could implement interventions to improve advance directive completion among underrepresented groups.

*RESPONSE: Disparities in AD completion among people who identified as Black or Hispanic suggest a need to adapt AD processes to better meet the needs of racially and ethnically historically underrepresented Veterans. In our discussion, we briefly review the advanced care planning literature. The existing evidence base for culturally sensitive advanced care planning interventions is quite limited and more high-quality intervention studies that address racial and ethnic disparities are needed. We point out this gap in our discussion. We edited the manuscript to note that “policy-based approaches to promote ACP could also play a role in AD completion.”*

Terminology should also be made consistent by using patients with COVID 19 throughout.

*RESPONSE: We have edited the manuscript to use this terminology consistently.*

Overall this is a valuable and well conducted study and addressing these points will enhance clarity rigor and impact.

*RESPONSE: Thank you.*
